# Clinical features of upper gastrointestinal endoscopy in 3146 patients: a 9-year retrospective cohort study in Zanzibar Archipelago, Tanzania

**DOI:** 10.4314/ahs.v23i2.45

**Published:** 2023-06

**Authors:** Li-Shuai Qu, Mariam Mohamed Gubi

**Affiliations:** 1 Digestive endoscopy center, Mnazi Mmoja Referral Hospital, Stonetown, Zanzibar Archipelago, Tanzania; 2 China Medical Team, Affiliated Hospital of Nantong University, Jiangsu province, China

**Keywords:** Upper gastrointestinal endoscopy, Helicobacter *Pylori*, Zanzibar

## Abstract

**Background/objectives:**

To investigate the demographic features, primary endoscopic findings, and the status of Helicobacter Pylori (H. pylori) infection of the enrolled subjects who underwent upper gastrointestinal endoscopy (UGIE) in the Zanzibar Archipelago, Tanzania.

**Methods:**

Between December 2013 and October 2021, a total of 3146 eligible participants were finally recruited in present retrospective cohort. Demographic information and endoscopic findings of each participant was retrieved. H. pylori infection was confirmed by rapid-urease test of gastric antral and body biopsies at endoscopy.

**Results:**

Among the recruited subjects, 1691 (53.76%) are females, remaining 1455 (46.24%) are males. The median age of this retrospective cohort was 40 years ranging from 8 to 97 years. The common identified endoscopic findings included gastro-duodenitis, normal endoscopic finding, peptic ulcer disease (PUD), esophagitis, esophagogastric varices, esophageal and gastric cancer, respectively. After adjustment for sex and age, a significant risk of gastric and/or duodenal ulcer (OR, 2.51; 95% CI, 1.82–3.48, P<0.001) and gastric cancer (OR, 3.49; 95% CI, 1.27–9.58, P=0.015) in H. *pylori* positive group was observed. Stratified analysis indicated a significant relationship between duodenal ulcer with younger age (adjusted OR, 0.98; 95% CI, 0.97–0.99, p = 0.002), and the presence of H. pylori (OR, 2.01; 95% CI, 1.12-3.91, p= 0.021).

**Conclusions:**

The present study revealed that gastro-duodenitis, PUD, and normal finding are the most common endoscopic diagnoses in Zanzibar. The presence of H. *pylori* is significantly associated with duodenal ulcer and gastric cancer.

## Introduction

Upper gastrointestinal endoscopy (UGIE) is considered one of the most effective procedures to provide valuable information for the diagnosis of gastroduodenal disorders, such as dyspepsia, persistent nausea and vomiting, dysphagia, chest or abdomen pain, hematemesis or melena, early satiety or anorexia, and so on^1^. The invention and development of endoscopy is a milestone in the structure of modern gastroenterology^2^. UGIE is an ideal method for identifying organic diseases of upper digestive tract and it has been widely used not only for diagnostic but also for therapeutic purpose^3^.

Tanzania, located in east Africa, is one of the poorest countries in the world. The Zanzibar archipelago, a semiautonomous region of the United Republic of Tanzania, comprises Unguja, Pemba, and many smaller islands, with a total population over 1.3 million^4^. Although UGIE has been proved to be safe and highly efficient, this service is not yet extensively available in developing countries including Zanzibar. To our knowledge, no studies published to date yet described the clinical characteristics and endoscopic findings in Zanzibar. Additionally, epidemiological data in chronic H. pylori infection are still lacking. In this retrospective cohort study spanning past 9 years, we aimed to investigate the demographic features, primary endoscopic findings, and the status of H. *pylori* infection of the subjects who underwent UGIE service in Zanzibar.

## Material and Methods

### Study design

Digestive endoscopy center of Mnazi Mmoja Referral Hospital was established in December 2013, and it is the only unit providing UGIE service in the whole Zanzibar. We retrospectively reviewed data from all medical in-patients and clinic out-patients who underwent UGIE examination at Mnazi Mmoja Referral Hospital in Stonetown, Unguja. This study which recruited adults and minors was conducted in accordance with the Helsinki Declaration and was approved by the research ethics committee at Mnazi Mmoja Referral Hospital in Stonetown, Unguja, Zanzibar Archipelago.

### Study population

Between December 2013 and October 2021, 3245 subjects underwent UGIE were enrolled in this retrospective cohort study. We excluded 24 patients who could not tolerate strong vomiting reaction during the procedure of UGIE examination. 29 subjects with severe food retention were removed because this condition might affect the observation and final diagnosis. We also excluded 46 cases of therapeutic endoscopies, such as those involving foreign body removal, polypectomy, or bleeding control. Consequently, a total of 3146 eligiblsubjects were finally entered into the analyses. Information on recruited patients was extracted from electronic endoscopy reporting system, including their sex and age at the time of UGIE, endoscopic diagnosis, and the result of rapid H.*pylori* test.

### Preparation and H. pylori test

UGIE was performed using the Olympus CV-70 videoscope system. A baseline hemoglobin and screen tests for HIV I and II, hepatitis C antibody, and hepatitis B surface antigen were done for all patients. All participants received standard pre-procedure preparation that included a fast at least 8 hours. Included patients had been given the sedation with 10% lidocaine throat spray before UGIE. H. *pylori* infection was determined by the rapid-urease campylobacter like-organism test on gastric body or antral biopsies at UGIE. Biopsies were routinely taken from lesions in the esophagus, stomach, and duodenum for further histological evaluation. Endoscopic diagnosis of each subject was recorded in electronic endoscopy reporting system.

### Statistical analysis

Data are presented as means ± SD, proportions, or median (range). To compare the values between the two groups, Pearson's χ^2^ or Fisher exact tests were performed for categorical variables and the student's t-test was used for continuous variables with normal distributions, respectively. Binary unconditional logistic regression models were used to estimate the odds ratios (ORs) of H. *pylori* infection associated factors and corresponding 95% confidence intervals (CIs). Potential confounders including age and sex were adjusted. All statistical tests were two-tailed, and a P value of less than 0.05 was considered statistically significant. Statistical analyses were performed using the Statistical Program for Social Sciences (SPSS 22.0 for Windows; SPSS, Inc., Chicago, IL).

## Results

### Demographic features of the study population

**Figure 1:**
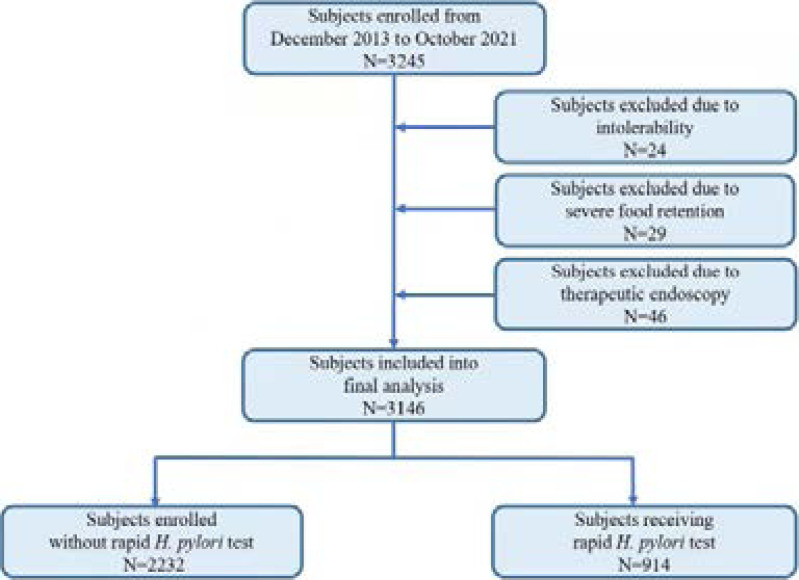
Flow chart of participants inclusion and exclusion of this retrospective cohort study

During the study period, a total of 3146 UGIEs were enrolled (flowchart of the study shown in Figure 1). Among these recruited subjects, 1691 (53.76%) are females, the remaining 1455 (46.24%) are males. The median age of this retrospective cohort was 40 years ranging from 8 to 97 years. The 20-39 years age group had the highest frequency of 1301 (41.35%) patients, followed by the 40-59 years age group with 1043 (33.15%) patients, while older than 80 years age group had the lowest frequency of 66 (2.10%) cases, respectively. The details of age and sex distribution are shown in Table 1.

**Table 1:** Patient characteristics

Characteristics	Number (N)	Frequency (%)
No. of patients	3146	
Male: female ratio	1455: 1691	46.24: 53.76
Age distribution (year)		
<20	251	7.98
20-39	1301	41.35
40-59	1043	33.15
60-79	485	15.42
≥80	66	2.10

### Year distribution of UGIE examinations

**Figure 2:**
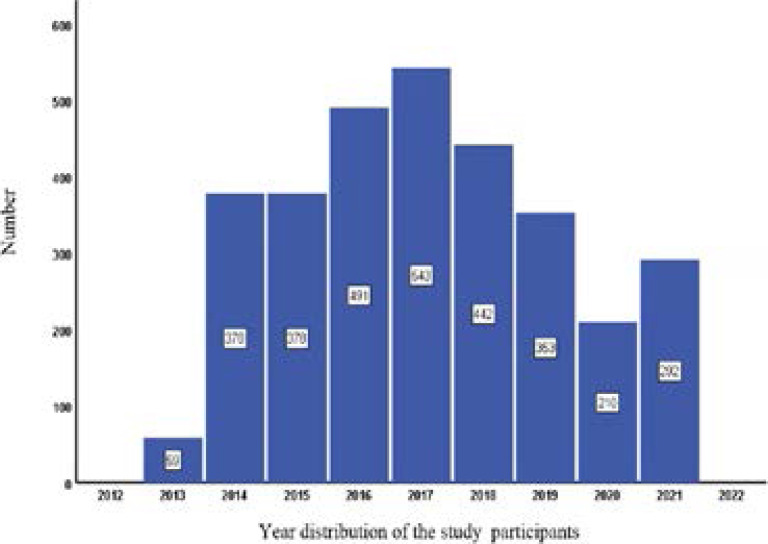
Year distribution of UGIE examinations in past 9 years

From December 2013 to October 2021, 3146 UGIEs distributed in nearly 9 years (shown in Figure 2). The highest frequency of 543 patients appeared in 2017, followed by 491 patients in 2016 and 442 subjects in 2015, and then 378 cases in 2014 and 2015, respectively. Compared with other years, only 210 patients received the UGIE because of the global pandemic of COVID-19.

### Primary UGIE findings

In our study, 1315 (41.80%) patients revealed gastro-duodenitis and this was the most common identified endoscopic finding, 953 (30.30%) of the 3146 subjects had normal endoscopic findings. Other positive UGIE findings included gastric and/or duodenal ulcer in 380 (12.08%) patients, esophagitis in 171 (5.44%) patients, esophagogastric varices in 111 (3.53%) cases. Histologically confirmed esophageal cancer and gastric cancer was diagnosed in in 81 (2.57%) patients and 44 (1.40%) subjects, respectively. Less commonly reported findings were polyps seen in 34 (1.08%) cases, sub mucosal tumor in 28 (0.89%) patients, and hiatal hernia in 20 (0.64%) patients. The frequency of less than 10 cases classified according to the UGIE findings was shown in Table 2.

**Table 2:** Primary UGIE findings (N=3146)

Endoscopic findings	Number (N)	Frequency (%)
Gastro-duodenitis	1315	41.80
Normal findings	953	30.30
Duodenal ulcer	244	7.76
Esophagitis	171	5.44
Gastric ulcer	121	3.85
Esophagogastric varices	111	3.53
Esophageal cancer	81	2.57
Gastric cancer	44	1.40
Polyps	34	1.08
Sub mucosal tumor	28	0.89
Hiatal hernia	20	0.64
Gastroduodenal compound ulcer	15	0.48
Cardiac cancer	11	0.35
Laryngeal and/or pharyngeal cancer	11	0.35
Esophageal achalasia	9	0.29
Esophageal ulcer	7	0.22
Mallory-Weiss Syndrome	4	0.13
Duodenal cancer	2	0.06

### Clinical characteristics of *H. pylori* positive and negative groups

Due to the shortage of H. pylori detection reagents, rapid urease test was successfully performed in only 914 subjects and therefore these were included into analysis. The major endoscopic diagnoses included normal findings which occurred in 297 (32.49%) patients, followed by gastro-duodenitis in 289 (31.62%), gastric and/or duodenal ulcer in 226 (24.73%), esophagitis in 78 (8.53%), gastric cancer in 22 (2.41%), and other diagnoses in 2. Among 914 patients, positive result indicated H. *pylori* infection in 453 (49.56%), while negative result in 461 (50.44%). The demographic data of the H. *pylori* negative and positive groups are listed in Table 3. There were no statistically significant differences in age and gender distribution. Compared with patients without H. *pylori* infection, after adjustment for sex and age at recruitment, unconditional logistic regression analyses showed a significant higher risk of gastric and/or duodenal ulcer (adjusted OR, 2.51; 95% CI, 1.82–3.48, P<0.001) and gastric cancer (adjusted OR, 3.49; 95% CI, 1.27–9.58, p=0.015) in H. *pylori* positive group. Meanwhile, absence of H. *pylori* demonstrated a statistical trend to normal endoscopic findings (adjusted OR, 0.59; 95% CI, 0.48–0.79, P<0.001). There was no significant association between the presence of H. *pylori* and the diagnoses of gastro-duodenitis and esophagitis.

**Table 3:** Characteristics of H. *pylori* positive and negative cases

Variable	H. *pylori* negative N= 461 (%)	H. *pylori* positive N= 453 (%)	Adjusted odds ratio* (95% CI)	*P*-value
Male: female ratio	226 (49.02):235 (50.98)	234 (51.66):219 (48.34)	-	0.426
Age (years), mean ± SD Primary endoscopic findings	41.54 ±16.67	42.00 ±17.86	-	0.685
Normal findings	176 (38.18)	121 (26.71)	0.59 (0.48-0.79)	<0.001
Gastro-duodenitis	158 (34.27)	131 (28.92)	0.79 (0.59-1.04)	0.095
Gastric and/or duodenal ulcer	77 (16.70)	149 (32.89)	2.51 (1.82-3.48)	<0.001
Esophagitis	43 (9.32)	35 (7.73)	0.81 (0.51-1.30)	0.389
Gastric cancer	5 (1.08)	17 (3.75)	3.49 (1.27-9.58)	0.015
Other diagnosis	2 (0.44)	0	-	-

* Adjusted for sex and age.

### Clinical characteristics between gastric and duodenal ulcer cases

Among all recruited subjects, 380 (12.08%) patients suffered gastric and/or duodenal ulcer. After excluding 15 patients with complex ulcer in stomach and duodenum, remaining 365 patients were divided into gastric and duodenal ulcer groups. There was no statistically significant difference in gender distribution (p=0.185). The average ages were 50.15±17.43 years in gastric ulcer group an43.55±19.28 years in duodenal ulcer group, respectively. The elder group had significant higher risk of gastric ulcer but lower risk of duodenal ulcer (adjusted OR, 0.98; 95% CI, 0.97–0.99, p=0.002). Of these 365 patients, 215 received H. *pylori* rapid urease test. In stratified analysis, 36 (54.55%) were positive for H. *pylori* infection in gastric ulcer group, while 106 (71.14%) were positive in duodenal ulcer group. The relationship between presence of duodenal ulcer and H. *pylori* infection was statistically significant (adjusted OR, 2.01; 95% CI, 1.12-3.91, p=0.021) (Table 4).

**Table 4:** Characteristics of gastric and duodenal ulcer cases

Variable	gastric ulcer N= 121 (%)	duodenal ulcer N= 244 (%)	Odds ratio (95% CI)	*P*-value
Gender				
Female	44 (36.36)	72 (29.51)	1.00 (reference)	
Male	77 (63.64)	172 (70.49)	1.37 (0.86-2.17)	0.185
Age (years), mean ± SD	50.15 ±17.43	43.55 ±19.28	0.98 (0.97–0.99) *	0.002
H. *pylori* test performed subjects (N)	66	149		
Negative	30 (45.45)	43 (28.86)	1.00 (reference)	
Positive	36 (54.55)	106 (71.14)	2.01 (1.12-3.91) ^†^	0.021

* Adjusted for sex

† Adjusted for sex and age

## Discussion

As mentioned previously, UGIE is a mini-invasive and convenient method that plays an important role in the diagnosis of upper gastrointestinal tract diseases^5^. Meanwhile, distinct clinical characteristics and outcomes of the UGIE have been reported in different geographical parts of the world^6^. The data in Zanzibar Archipelago remain unknown. This research represents the first ever study on UGIE findings from Zanzibar, Tanzania. Our data documented the demographic characteristics, endoscopic findings, and H. *pylori* status in a national hospital of Zanzibar.

Our results showed subjects under 60 years old made up the majority of enrolled group and the proportion of male and female was balanced. All cases were evenly distributed in 9 years except relatinumber in 2020 for the influence of COVID-19 pandemic. Similar to the result of previous studies in Ghanaian^7^,^8^, the presence of gastro-duodenitis was the common endoscopic finding among including patients. The data of normal endoscopic findings was lower than most past similar studies published in Zambia, Malawi, and Uganda across Africa^9^-^11^. From our data, the prevalence of PUD was lower to the result (15.7%) from a similar study performed in Denmark between 1993 and 2002^12^. Duodenal ulcer was detected more frequently than gastric ulcer among our study population in the ratio of more than 2:1. In stratified analysis, compared with gastric patients, duodenal ulcer group had a significant lower age and higher incidence of H. *pylori* infection. This trend was consistent with results from other studies worldwide. The percentage of esophageal varices detected in Zanzibar was higher than a similar retrospective review of the records conducted in Ghana^13^. Two retrospective studies reported in Tanzania had also documented comparably high rates of esophageal varices among patients with upper gastrointestinal bleeding (41%-52%)^14^,^15^. We speculated that the high prevalence of esophageal varices might be attributed to hyper-endemic schistosomiasis in this region^16^,^17^. Upper gastrointestinal malignancies detected in this study mainly included esophageal cancer and gastric cancer. The majority of them underwent endoscopy due to dysphagia or upper gastrointestinal bleeding.

H. *pylori* is a common bacterium, and infects approximately 50% of the world's population^18^. The prevalence of H. pylori infection is highly variable across different countries^19^. In this retrospective study, the ratio of H. *pylori* infection in our sample of subjects is also evaluated. Only part of the study patients has access to receive rapid-urease testing due to the shortage of H. *pylori* detection reagents in Zanzibar. Among 914 patients, H. pylori infects about half of the population. The clinical features of H. *pylori* vary from asymptomatic carriers to gastrointestinal carcinoma^20^. It has been reported that up to 50% of the gastric ulcers and 80%-90% of the duodenal ulcers are related with H. *pylori* infection^21^,^22^. Our data indicated that more than half of patients were positive for H. *pylori* infection in gastric and/or duodenal ulcer group. Compared to patients without H. *pylori* infection, H. *pylori* positive subjects suffered a significant higher risk of gastric and/or duodenal ulcer In subgroup analysis, the percentage of H. *pylori* positive in gastric ulcer and duodenal ulcer group was 54.55% and 71.14%, respectively. Further analysis demonstrated the incidence of duodenal ulcer with H.*pylori* positive is significantly higher than that of gastric ulcer. As we all known, H.*pylori* is classified as group I carcinogen^23^. Nearly 75% of the global gastric cancer burden and 5.5% of malignant tumor worldwide are attributable to H pylori-associated inflammatory response and injury^24^,^25^. In this study, we also investigate the risk of H. *pylori* in the development of gastric cancer. After adjustment for sex and age, a strong relationship between H. *pylori* and gastric cancer was observed.

To our knowledge, this is the first retrospective study investigated the clinical features and outcomes of UGIE over a period of 9 years in Zanzibar. There are also some limitations that should be considered. First, we could not retrieve the information and clinical symptoms of patients, the association between living styles and indications for UGIE and positive endoscopic findings could not be assessed. Second, a major limitation of present study is lacking of a gold standard about assessing the accuracy of UGIE. The possibility of false positive and false negative results still exists. Third, in resource limited settings, we could not collect data on use of common anti-H. *pylori* regimen during peri-UGIE period. Even considering false negative results of H. *pylori* caused by such medicine, our statistic still confirmed the high risk between H. *pylori* and duodenal ulcer and gastric cancer. It tends to underestimate rather than overestimate the risk of H. *pylori* infection. Finally, as a retrospective cohort study, it is difficult to reach a firm conclusion and a large-scale, well-designed study with long-term follow-up should be conducted in future.

## Conclusion

In conclusion, the present study revealed that gastro-duodenitis, peptic ulcer disease, and normal finding are the most common endoscopic diagnoses. Due to presence of H. *pylori* infection in significant number of duodenal ulcer and gastric cancer patients, completed eradication treatment is strongly recommended according to guideline in Zanzibar.
